# Bacterial Biofilms Utilize an Underlying Extracellular DNA Matrix Structure That Can Be Targeted for Biofilm Resolution

**DOI:** 10.3390/microorganisms10020466

**Published:** 2022-02-18

**Authors:** Steven D. Goodman, Lauren O. Bakaletz

**Affiliations:** Center for Microbial Pathogenesis, Abigail Wexner Research Institute at Nationwide Children’s Hospital, The Ohio State University College of Medicine, Columbus, OH 43205, USA; lauren.bakaletz@nationwidechildrens.org

**Keywords:** biofilm, disruption, Z-DNA, humanized monoclonal antibody, tip-chimer peptide, chinchilla, nucleoid associated proteins, PMN, NET

## Abstract

Bacterial biofilms contribute significantly to the antibiotic resistance, pathogenesis, chronicity and recurrence of bacterial infections. Critical to the stability and survival of extant biofilms is the extracellular DNA (eDNA)-dependent matrix which shields the resident bacteria from hostile environments, allows a sessile metabolic state, but also encourages productive interactions with biofilm-inclusive bacteria. Given the importance of the eDNA, approaches to this area of research have been to target not just the eDNA, but also the additional constituent structural components which appear to be widespread. Chief among these is a ubiquitous two-member family of bacterial nucleoid associated proteins (the DNABII proteins) responsible for providing structural integrity to the eDNA and thereby the biofilm. Moreover, this resultant novel eDNA-rich secondary structure can also be targeted for disruption. Here, we provide an overview of both what is known about the eDNA-dependent matrix, as well as the resultant means that have resulted in biofilm resolution. Results obtained to date have been highly supportive of continued development of DNABII-targeted approaches, which is encouraging given the great global need for improved methods to medically manage, or ideally prevent biofilm-dependent infections, which remains a highly prevalent burden worldwide.

## 1. Introduction

Biofilms are communities of microorganisms enshrouded in an extracellular matrix. The matrix is primarily self-made although bacteria are known to also include environmental or host components [1,2]. The matrix both protects and creates permissive conditions that alter the metabolism, physiology and gene expression of resident microorganisms to allow them to both exist in a mostly sessile state and resist environmental challenges and thereby improve survival [3]. Indeed, biofilms can be more than 1000-fold more tolerant to antibiotics than their free-living planktonic counterparts. The matrix material consists of multiple biomolecules including proteins, lipids, polysaccharides and nucleic acids [3]. Constituent matrix molecules vary amongst bacterial species but extracellular DNA (eDNA) appears omnipresent. Forty years ago, the DNABII family of proteins, one of the members of the nucleoid associated proteins (NAPS) that create and or stabilize DNA architectures within the bacterial cell was discovered to also exist extracellularly. Multiple investigators were able to recapitulate this result for various species of bacteria [4,5,6,7,8,9,10]. The DNABII family is novel in that it is the only NAP that is ubiquitously expressed among all eubacteria; at least one allele has been identified in every sequenced bacterial species [11]. As will be described below, eDNA and DNABII proteins are necessary components of the underlying eDNA-dependent biofilm matrix. In order for multiple bacterial species to create an inclusive interactive community within a biofilm, the protective extracellular matrix must be sufficiently compatible to all biofilm-resident bacteria. We posit that the most likely means to this end, is to have a common matrix infrastructure that we have shown incorporates at least eDNA and DNABII proteins.

The following is a review of what is known about this eDNA-DNABII dependent matrix, including its structure and how the knowledge of this structure can be used to undermine biofilms to release resident bacteria back into a vulnerable state.

## 2. eDNA

Bacteria have been known to release DNA since Avery, Mcleod and McCarty showed eDNA was responsible for transformation nearly 80 years ago [12]. Indeed, many bacterial species are capable of being transformed, i.e., have the ability to take up eDNA and incorporate the genetic information. Bacteria typically need to be in a state termed *competence* in order for transformation to occur and thus there are whole apparatuses that are specific to individual species to accomplish this task, with the caveat that these systems are designed to be selective for homospecific eDNA. Additionally, while the evolutionary role of transformation is unknown, transformation is a very potent means for horizontal gene transfer. Importantly, this process is enhanced in a biofilm [13,14]. Thus, the presence of released eDNA is likely to be more evolutionarily driven than a random event. Indeed, in one case, competent nontypeable *Haemophilus influenzae* was found to release its chromosomal DNA, in part, through the competence-induced binding protein, ComE (an outer membrane pore through which the type IV pilus is also extruded), the same protein that was originally found to be essential to take up eDNA during transformation [15].

It was clear from the earliest investigations, however, that eDNA would likely possess a structural role in the biofilm matrix. In the 1950s, microbial mats, a type of environmental biofilm, were discovered to incorporate DNA as one of their matrix materials [16]. Later, others showed that DNase could reduce the ‘stickiness of slime’ of some Halobacter communities as well as those formed by *Pseudomonas aeruginosa* [16,17]. These works established that eDNA was a common constituent of bacterial biofilms. In a 2002 *Science* paper [18], Cynthia Whitchurch and coworkers introduced evidence that bacterial biofilms formed by *Pseudomonas aeruginosa* were reliant on eDNA during development. Indeed, DNase added with bacteria prevented biofilm formation. However, as the biofilm matured, DNase became less effective at disrupting the extant biofilm. Multiple examples in other single-species biofilms followed, with the caveat that in every case, biofilm disruption became recalcitrant to DNase with time [3]. Given this limited experimental perspective, it was thus generally accepted at the time that eDNA was important for biofilm formation, but once formed, eDNA was no longer critical for matrix integrity.

During this same period of time, two important observations were made. First, it became clear that there was another critical source of eDNA, namely that originating from the mammalian host. Indeed polymorphonuclear leukocytes (PMNs), the sentinels of innate immune system, can go through a transformation wherein their chromatin is released to create a Neutrophil Extracellular Trap (NET). Briefly, induction of this process, termed NETosis, as the result of either infectious virulence factors or immune stimulation can cause PMNs to release their eDNA in a net-like array (hence the name) to either ensnare and kill individual bacteria or to cordon off biofilm proliferation. With regard to the former, NET eDNA is used to concentrate associated antimicrobial factors (e.g., histones) to kill bacteria trapped in these NETs. While this strategy has limited effectiveness on biofilms per se, it does prevent bacteria from leaving the biofilm and thus pathogen proliferation. Second, investigators began to notice that the bacterial eDNA possessed a lattice-like structure [19,20] regardless of the source, i.e., whether the eDNA was derived from the mammalian host to restrict biofilm growth (as mediated by PMN NETosis) or from the bacterium to build its biofilm.

## 3. DNABII Proteins

While bacteria are devoid of histones, they possess various proteins that are known to manipulate the gross structure of bacterial chromatin, termed nucleoid associated proteins or NAPs. While some NAPs are mostly conserved across eubacteria, only the DNABII family is ubiquitous, with at least one allele present in the genomes of all examined eubacteria [11]. This two member family [Integration Host Factor (or IHF) and HU (a histone like protein originally isolated from *E. coli* strain U93)] consists of homologous proteins whose active form is a homo- or heterodimer [21]. While both members of the family have similar DNA binding properties, IHF also binds to a specific consensus sequence while HU proteins appear to have no DNA sequence specificity. The proteins are small, typically < 100 amino acids, and strongly basic to facilitate electrostatic interactions with DNA. Probably the most important feature of DNABII binding to DNA is the capacity of the protein to bend DNA upon binding or bind to bent DNA with higher affinity over unbent DNA, as the energy of binding is strongly facilitated by a bent conformation. With regard to the latter, DNABII proteins are known to bind with high affinity to Holliday Junctions (HJs), recombination intermediates that are four-armed DNA structures that resemble a cruciform where any two adjacent arms mimic a single double stranded bent DNA molecule and can thus be bound by a DNABII protein with high affinity [22,23,24,25]. Indeed, the lattice-like structure of biofilm eDNA visually resembles an array of contiguous HJs.

**Figure 1:**
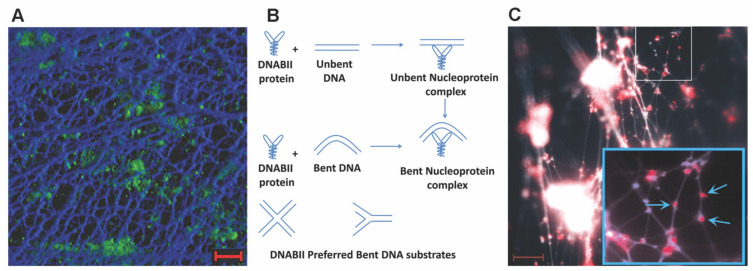
Localization of a DNABII protein at vertices of bent eDNA within an in situ biofilm. (**A**) Immunofluorescent image of a biofilm formed in the middle ear of the chinchilla (21 days after challenge with non-typeable *Haemophilus influenzae*). Fine, widely spaced dsDNA strands are labeled with DAPI and appear blue in this image, whereas non-typeable *H. influenzae* are labeled with FITC-conjugated antiserum directed at a surface-exposed protein and appear green. Marker bar = 5 µm. (**B**) DNABII family members have a stronger preference for binding to bent DNA—a generalized scheme. (**C**) Immunohistochemical labeling of IHF within an NTHI biofilm formed in vivo. The strong labeling of each vertex where individual strands of DNA cross (arrows) must be noted. FITC, fluorescein isothiocyanate; IHF, integration host factor; NTHI, non-typeable *Haemophilus influenzae*. Reprinted from Ref. [9].

Importantly, DNABII proteins have been found in great abundance extracellularly by multiple investigators [4,5,6,7,8,9,10]. Given that there is an ample steady state level of eDNA, and the common lattice structure was ubiquitous, the disposition of the DNABII proteins was examined in an in vivo model of otitis media (OM) where NTHI was introduced into the middle ear of a chinchilla resulting in a biofilm disease state. Intriguingly, when examined by an immunohistochemical approach using antisera directed to DNABII proteins, a member of the DNABII family was observed positioned at the vertex of each crossed-strand of bacterial eDNA within the lattice of the NTHI biofilm (Figure 1), which suggested that they may serve a role as a structural linchpin. These results have been recapitulated in vitro and in vivo (both in animal models and ex vivo samples of human biofilm disease) [9,25,26,27,28,29].

Interestingly, other NAPs appear to fail to contribute to the structural integrity of biofilms. In the most comprehensive study to date with NTHI [30], where all NAPs were found extracellularly in great abundance, only IHF and HU were found to contribute to the structural integrity of the biofilm. 

## 4. Mechanisms of eDNA and DNABII Release

Of all the topics discussed here, the mechanisms that eubacteria use to release these critical biofilm components is likely to vary the most amongst genera and species, and release of DNA will be no different. For example, in streptococci competent for transformation, there is a quorum sensing induced phenomenon termed fratricide, where bacteria induce autolysis, thus releasing DNA and the rest of the bacterial contents as common goods for the surviving bacteria [31]. To date, the best characterized release mechanism was determined for NTHI. For this bacterium, a small subpopulation of NTHI actively releases its own DNA into the extracellular environment [15], wherein DNABII proteins (but not other nucleoid associated proteins or NAPs) are responsible for the three-dimensional lattice-like array that results and which provides structural stability for the biofilm and harbors the biofilm-resident bacteria and all the other constituents of the EPS [30,32,33,34,35]. This release of eDNA and DNABII proteins into the biofilm by NTHI occurs via a novel mechanism wherein this bacterium uses of both remnants of a stolen Type 4 Secretion System (T4SS) and the machinery integral to expression of the Type IV twitching pilus, which includes proteins specifically expressed during competence [15].

## 5. Immunological Depletion of DNABII Disrupts Biofilms In Vitro

Via the use of a polyclonal antiserum directed against IHF produced by *E. coli*, we learned that incubation of a pre-formed NTHI biofilm with this antiserum resulted in collapse of that 3D structure [9]. In a series of studies that followed, we expanded our understanding of the molecular mechanisms that lead to this structural collapse that was further found to be rapid, dose-dependent, highly specific and did not require direct contact between the antibodies and the biofilm matrix. Further, we showed that this rapid significant structural collapse of the biofilm was predicated by an equilibrium shift, wherein the antibodies bound free DNABII proteins on their DNA-binding surface and thereby prevented rebinding [36]. Via collaboration with other investigators, in addition to ongoing studies in our own laboratories, we demonstrated that what we had shown regarding the induced collapse of the NTHI biofilm was also observed with biofilms formed in vitro by multiple additional human pathogens [28,37,38,39,40], including the other two predominant otopathogens, *Streptococcus pneumoniae* and *Moraxella catarrhalis*. One of our most intriguing observations was that synonymous with the collapse of these biofilms, the formerly biofilm-resident bacteria were now released into the surrounding environment in a phenotypic state that to date possesses the highest sensitivity to antibiotics [36,41,42]. This phenomenon has been observed by others [43,44,45,46,47] and thereby is an area of active investigation in many laboratories. We too have begun to explore this distinct phenotype for NTHI via proteomics, metabolomics and ongoing transcriptional profiling, as we concur that having a greater understanding of this ‘newly released’ (or NRel) phenotype may indeed prove highly fruitful for the development of improved clinical management approaches that target these NRel bacteria for OM and other diseases wherein biofilms contribute greatly to pathogenesis, chronicity and recurrence. 

## 6. Immunological Depletion of DNABII Disrupts Biofilms In Vivo: Clinical Relevance and Pre-Clinical Data

Whereas our work to this point was interesting as an in vitro phenomenon, we needed to understand if the presence of a bacterial eDNA lattice (with associated linchpin structural DNABII proteins) was also a clinically relevant observation. Toward this goal, we recovered a number of diverse clinical specimens to determine if we could demonstrate the presence of a bacterial eDNA lattice with associated DNABII proteins in specimens recovered from human disease sites. To date, we have reported that these intricate lattices are indeed present in: post-C-section wound infections [48]; sputum recovered from children with Cystic Fibrosis (CF) [27]; the exudate recovered from the auditory canals of children with post-tympanostomy tube otorrhea [29]; and in the middle ear fluids of pediatric OM with effusion (OME) patients [49]. These results not only show the ubiquity of this phenomenon, regardless of the origin of biofilms, but also demonstrates that bacteria resident within the biofilm rely on this underlying structure for survival, which thus provides us with a universal target regardless of which species are constituents of a pathogenic biofilm.

With the strong likelihood of clinical relevance established, we wondered if we could begin to develop a therapeutic strategy and/or a prevention strategy that could leverage this understanding of biofilm structural integrity to prevent biofilm formation in the middle ear (or other body site) or to resolve existing and perhaps long-standing biofilms [as present within the middle ears of children with Chronic Suppurative Otitis Media (CSOM) or in the lungs of those with CF, for example]. According to our model, antibodies that would bind the DNABII proteins and prevent their subsequent re-binding to eDNA would make good therapeutic candidates. As a first step, we epitope mapped a DNABII protein to determine which domains could be defined as protective, inclusive of those that are required for DNA binding. Indeed, antibodies that bound the DNA binding ‘tips’ were deemed most protective. We used this information to develop a murine monoclonal antibody directed against the DNA-binding ‘tips’ of the DNABII proteins [50]. Via a series of studies, we have been able to demonstrate that this epitope-targeted monoclonal antibody could both eradicate existing biofilms from nasal packing material [51], as well as inhibit the formation of bacterial biofilms on that commonly used surgical site packing material [52]. Further, we utilized three unique animal models of human disease to determine that we could mediate rapid resolution of an osteolytic infection due to *Aggregatibacter actinomycetemcomitans* in rats [53], a lung infection in mice due to *Pseudomonas aeruginosa* [50] and experimental OM due to NTHI in a chinchilla [54]. We were also able to use a chimeric synthetic peptide modeled after the two DNA-binding tips of a DNABII protein (‘tip-chimer peptide’) to actively immunize chinchillas transcutaneously and prevent development of NTHI-induced OM in a viral-bacterial superinfection model (72% vaccine efficacy when delivered with the adjuvant dmLT) [55]. To address concerns about the potential to induce dysbiosis of the gut flora following active immunization with a vaccine antigen directed against a biofilm constituent (given that there are many ‘good’ biofilms on and in the human body as well), we immunized cohorts of chinchillas both via an subcutaneous and transcutaneous route with the same tip-chimer peptide to compare their gut microbiome to those that had been treated with the standard-of-care for OME (e.g., amoxicllin/clavulanic acid). We found that whereas orally administered amoxicllin/clavulanic did indeed rapidly induce dysbiosis of the gut flora, as expected, neither route of immunization with the DNABII-directed immunogen did so [56] which suggested that the induced antibodies did not likely transude onto the non-inflamed (non-diseased) mucosa that lined the GI tract with beneficial biofilms in these animals. 

## 7. eDNA-Dependent Structure Relies on HJs

While the exact structure and mechanisms of release of the eDNA remain elusive, much progress has been made. First, the cross strands of the lattice appear to both visually and functionally resemble HJs. Indeed, Devaraj et al. [35] was able to show that the DNABII proteins could be replaced with another HJ stabilizing protein, RuvA. Likewise, antibodies that recognize HJ structures also bound with high affinity to the same vertices. To further verify that these structures were functional orthologues, RuvA-stabilized biofilms were supplemented with RuvB and RuvC. Intracellularly, RuvA, RuvB and RuvC are part of complex that binds to HJs and cleaves them to complete homologous recombination, where RuvA binds to bona fide HJs, RuvB acts as a helicase to promote branch migration and RuvC binds to the RuvAB complex cleaving the HJ. As the cross stranded structures sufficiently resembled HJs, the RuvABC complex was able to cleave these structures which resulted in disruption of the biofilm. These results show that the vertices of the eDNA are functional orthologues of HJs, but more importantly, that they are necessary for eDNA-dependent biofilm integrity as well as the target of DNABII structural stabilization.

## 8. eDNA Is Important throughout Biofilm Development but Recalcitrant to Degradation by Various Nucleases

As Whitchurch and others observed, DNase which is a relatively non-specific nuclease, can readily prevent biofilm formation, however, as the biofilm matures its effectiveness wanes on extant biofilms. Indeed, uropathogenic *E. coli* and NTHI both become completely recalcitrant to DNase after 24 h of biofilm development. However, anti-DNABII mediated disruption was shown to be effective for highly mature biofilms (2 weeks; [36]) and yet still require eDNA [37]. How can these two disparate results be reconciled? Although some researchers believed that eDNA was only important for early biofilm formation, others examined various nucleases with varied DNA substrate specificities with the hope that the failure of DNase dependent disruption of mature biofilms was due to the limitations of DNase and not the lack of matrix dependent DNA. The most effective of these is NucB [57] and [58]. NucB is an endonuclease from a marine strain of *Bacillus linchenformis* and likely has a higher specific activity than bovine pancreatic DNase [57]. Despite this, NucB’s ability to disrupt some biofilms, even at 18 h of development, is neither universal nor complete. This outcome is in contrast to that achieved by treatment with anti-DNABII, which acts in a dose-dependent manner on all biofilms tested to date and can reduce biofilms to a monolayer regardless of ‘age.’ Likewise, HJ resolvases like RuvABC and RusA also appear to be agnostic against bacterial biofilms. Taken together, these results reinforce the hypothesis that eDNA matures into a nuclease-resistant state, except at the HJ-like vertices which while stabilized and occluded by DNABII are vulnerable to HJ resolvases. 

## 9. The Nature of the Nuclease-Resistant Structure of Biofilm eDNA

While the vertices of the eDNA possess an HJ-like structure, how does the bulk of the remaining eDNA fibers become resistant to nuclease activity? What other properties of eDNA are important? Others have shown that, at least in some in vitro biofilms, there are G quadruplexes [59]. These structures are sequence specific, requiring Hoogsteen base pairing, one hallmark of which are bases in the anti-syn conformation (Watson–Crick bases are in the anti–anti conformation), which allows changes in the hydrogen bond donors and acceptors and the proximity between C1′ carbons to be shorter between complementary DNA strands (although the base pairing partners, A-T and G-C do not change [60]). In this work, Seviour et al. [59] extracted eDNA from *Pseudomonas* biofilms under conditions where they attempted to preserve eDNA structure. This allowed them to examine the extracted eDNA by NMR and observe the Hoogsteen base pairing. Due to the novel structure, the authors returned to native biofilms and via immunofluorescence, showed the presence of G4 quadruplexes. How widespread these structures are, and whether they could contribute to the nuclease resistance of biofilms, remains to be determined.

More recently, we and another group nearly simultaneously reported the existence of Z-DNA conformations in the eDNA of biofilms [61,62]. While the first group [61] showed the ubiquity of eDNA in the Z-form on clothing, we conducted a more extensive investigation as to the origin, presence and role of Z-DNA within biofilms [62]. In Buzzo et al., we examined biofilms for their lability to DNase at seeding, but protracted stability as biofilms matured. We found that regardless of species, as bacterial biofilms matured, a greater proportion of eDNA became shifted into the Z-form, the only form of DNA known to be resistant to nucleases. Indeed, all but a few proteins bind exclusively to just B-DNA and not Z-DNA. Importantly, this Z-DNA phenomenon was ubiquitous even in ex vivo biofilm samples that were polymicrobial in nature. We then used agents that shifted the equilibrium of DNA from either B to Z, or Z to B forms, but failed to affect bacterial growth. Chloroquine, an intercalating agent that drives DNA into the B state, caused biofilm disruption even in polymicrobial ex vivo biofilm samples highlighting this pervasive and common eDNA structure regardless of bacterial species. Cerium Chloride, a large multivalent cation that drives DNA into the Z-state caused enhanced development of biofilms. Taken together, these data suggest that driving the eDNA into the Z state facilitates biofilm formation. 

In this same work, we then went further to show that conversion of B DNA to the Z-form also had consequences on host innate immune defenses as even just biofilm proximity to a bacterial biofilm had notable deleterious effects on the host, and specifically on NETosis. One of the first innate immune responders are PMNs, that are prone to release their DNA when they encounter bacterial pathogens. This transformation of PMNs into a NET provides a two-pronged attack on pathogens. First, the eDNA acts to entrap and kill individual bacteria or cordon off biofilm proliferation. Second, antimicrobial substances, e.g., histones, take advantage of their ability to bind to eDNA to enhance their directed diffusion towards the ensnared bacteria/biofilm. For the NET strategy to be successful, the eDNA needs to be in the native B-form as random diffusion reduces the targeted effectiveness of these NET-associated bactericidal agents and causes collateral damage to the host. We found that ex vivo biofilm sections derived from the middle ear of an infected chinchilla showed both a biofilm adhered to the epithelial surface and an extensive area that contained PMNs and NETs attempting to cordon off biofilm proliferation. When examined by immunofluorescence for B and Z-DNA, the 11-day-old biofilm contained exclusively Z-DNA, whereas the eukaryotic eDNA within the PMN NETs showed a gradient of proportionally higher density of Z-DNA conformation as the NET eDNA was located closest to the biofilm. Indeed, the NET eDNA extended 10 times further than the thickness of the biofilm and Z-DNA could be observed throughout the NET, except for the most distal regions wherein newly released NET eDNA was still in the B-form. Next we showed that merely adding DNABII proteins to NETs converted the native B-DNA into the Z-form. We then tested whether NETs maintained their killing function in the presence of DNABII proteins. Importantly, when DNABII proteins were added to NETs, the ability of the NETs to kill bacteria was completely abrogated. Taken together, these observations provide strong support for the hypothesis that as biofilms mature, they not only protect resident bacteria from nucleases and antimicrobials, but they also take on an offensive role, wherein released DNABII proteins convert NET eDNA from B to Z and this conformational change inactivates NET protective functions. Such steps are likely to be a prelude to programmed dispersal events, where biofilms release some of their resident bacteria to seed new locations and proliferate new biofilms.

While it remains to be determined how to reconcile both the G quadruplexes and Z-DNA as coincident matrix structures, since there is evidence that either abrogation of G quadruplexes or changes in the ratio of B and Z affect the viscoelastic properties of the biofilms that were examined, it is known that like G quadruplexes, in Z-DNA some of the bases are in the anti-syn configuration. Therefore, it is possible that these structures may be part of an equilibrium that exists in the eDNA of the matrix under different conditions and provide more clues as to how these structures form and how they may be undermined.

## 10. Inclusivity vs. Exclusivity and Future Work

eDNA and the DNABII proteins are common to the biofilms formed by all eubacteria, giving individual species the ability to form inclusive polymicrobial biofilms with those that share the same compatible underlying matrix material and structure. The fact that this matrix and its structure in mammalian hosts is used to both defend the resident biofilm bacteria and to undermine the host immune systems shows that the matrix has evolved to become recalcitrant to the host.

How then to reconcile all of the many other diverse biofilm matrix materials that are species specific? Others have identified a lengthy list of matrix materials including polysaccharides and proteins that are prominent in the biofilms formed by specific bacteria [3]. We posit that these materials are either designed to be compatible with the eDNA infrastructure, meaning that they are independent or alter (e.g., stabilize or specialize) the native eDNA-dependent structure, or that they supplant the structure with some other matrix material, and/or destabilize the structure; perhaps as a prelude for dispersal. In the former case, there are substances that support Z-DNA (e.g., biogenic amines) that could facilitate biofilm structure; these could be used to improve the kinetics of biofilm formation. Alternatively, there are proteins known to bind to Z-DNA that could be used to stabilize the Z-DNA structure. With regard to the latter, if bacteria need to be exclusive either to their own species or just a few select compatible species, they may have evolved alternative pathways that are eDNA independent in favor of novel matrix materials that only permit certain bacteria from entering a biofilm. This strategy could be used, e.g., by a pathogen to induce dysbiosis so that the infecting pathogen can establish itself among a consortia of bacteria in order to favor itself.

Future work in our laboratories will cover several major categories. First, we would like to determine the exact structure of the eDNA-dependent matrix and how it is assembled. Second, we are interested in describing the factors that drive this structure as well as those forces that disfavor this matrix development. The answer to each of these questions could provide additional targets for biofilm disruption. Likewise, these same features could conceivably be used to develop methodologies that drive the formation of beneficial biofilms and the colonization of probiotics [63]. Third, we seek to determine how the environment contributes to formation of the eDNA-dependent matrix. Do external sources of DNA contribute to biofilm formation? How does this affect the ‘decision tree’ of the resident bacteria to form a biofilm? Finally, how do these structures affect the biology of the biofilm? How to they affect the protective function of the matrix, e.g., against antimicrobials. While there are many questions to yet answer, it has become clear that eDNA is not just a constituent of the biofilm matrix, it is the underlying structure that all bacteria rely on to form their 3D communities. 

## Data Availability

Not applicable.
